# Hemp Stem Epidermis and Cuticle: From Waste to Starter in Bio-Based Material Development

**DOI:** 10.3390/polym14142816

**Published:** 2022-07-11

**Authors:** Maria Tommasina Pecoraro, Cristina Mellinas, Simona Piccolella, Maria Carmen Garrigos, Severina Pacifico

**Affiliations:** 1 Department of Environmental, Biological and Pharmaceutical Sciences and Technologies, University of Campania ‘Luigi Vanvitelli’, Via Vivaldi 43, 81100 Caserta, Italy; mariatommasina.pecoraro@unicampania.it; 2 Analytical Chemistry, Nutrition and Food Sciences Department, University of Alicante, 03080 Alicante, Spain; cristina.mellinas@ua.es (C.M.); mc.garrigos@ua.es (M.C.G.)

**Keywords:** hemp stem epidermis, hemp cellulose, agro-waste valorization, green biocomposite films, chemical characterization, reinforcing fillers

## Abstract

Nowadays, hemp farmers are facing an urgent problem related to plant stem disposal after seed harvesting. In this work, the commonly discarded epidermis and cuticle of hemp stems were valorized, turning them towards a sustainable recycling and reuse, contributing to the circular economy concept. Cellulose deprived of amorphous regions was obtained by a green process consisting of an ethanolic ultrasound-assisted maceration followed by mild bleaching/hydrolysis. The obtained hemp cellulose was esterified with citric acid resulting in a 1.2-fold higher crystallinity index and 34 °C lower T_g_ value compared to the non-functionalized hemp cellulose. Green innovative biocomposite films were developed by embedding the modified cellulose into PLA by means of an extrusion process. The structural and morphological characterization of the obtained biocomposites highlighted the functionalization and further embedment of cellulose into the PLA matrix. Attenuated Total Reflectance–Fourier Transform Infrared spectroscopy (ATR-FTIR) results suggested physical and chemical interactions between PLA and the organic filler in the biofilms, observing a homogeneous composition by Field Emission-Scanning Electron Microscopy (FESEM). Moreover, some increase in thermal stability was found for biocomposites added with 5%wt of the hemp cellulose filler. The obtained results highlighted the feasible recovery of cellulose from hemp stem parts of disposal concern, adding value to this agro-waste, and its potential application for the development of novel biocomposite films to be used in different applications.

## 1. Introduction

Nowadays, the management of residues coming from the agricultural sector has become a global matter of concern [1]. Europe has been a pioneer in encouraging a number of regulations and guidelines, aimed at minimizing the generation of agro-wastes and/or their re-use and valorization [2]. In this scenario, agro-wastes processing for recovering bioactive compounds or developing innovative and green bio-based materials is in full agreement with the motto of the circular economy. This considers the reuse and recycling of residues as a starting point for building a network of industrial strategies, leading to the definition of a mechanism for a sustainable future [3].

In Italy, industrial hemp is experiencing a revival as a polyfunctional crop since the publication of the 242/2016 Law by the Italian Ministry of Agriculture, Food and Forests [4]. This fact has led to a renewed investigation of the plant, deepening into its agronomic and phytochemical aspects, also based on available genotypes and their potential applications [5,6,7,8]. Monoecious hemp plants are commonly cultivated for seed production and food/nutraceutical purposes, whereas dioecious genotypes are considered more suitable for fiber exploitation [9]. Beyond genotypes, harvesting time and plant density in agronomic practices are determinants in defining the hemp products’ quality and they should be optimized to preserve fiber quality during grain ripening [10]. Hemp stems represent an under-used material and important waste, considering the only hemp seed supply chain, generating environmental and economic problems as a consequence of their disposal.

New potential applications to obtain biodegradable and biosustainable materials from hemp stems have contributed to considering hemp as a very promising renewable resource [11]. The use of hemp fibers as reinforcement in composite materials has received increasing attention due to their lower density, renewability, unlimited availability, and higher strength compared to conventional fibers. When combined with polymer matrices, biocomposites with improved mechanical performance as well as renewable, biodegradable, and recycling properties can be obtained [12,13,14,15], which can be used for packaging purposes in the food and cosmetic sectors, fulfilling the actual challenges of products’ preservation, sustainability, and biodegradability. Recently, composite materials based on hemp fibers as reinforcing agents and different thermoplastic polymer matrices, such as polypropylene (PP), bicomponent PP/polyester (BI), and recycled polyester (PES), were developed, obtaining the highest tensile strengths for composites containing 30% to 50% hemp fibers [16].

Extractable cellulosic materials have been proven to be more advantageous than synthetic ones as reinforcing fillers, due to their excellent physicochemical characteristics. In this work, the epidermis and cuticle from seed-type (monoecious) hemp stems have been investigated for the first time as a renewable source of cellulose, turning the studied wastes towards sustainable recycling and reuse. These plant components are commonly discarded unless unretted fibers are employed. Furthermore, when retting is not applied, the fiber tissue remains attached to the cuticle and epidermal tissues. For technical applications, hemp bast fibers (mainly in the paper and textile industries) and woody core fibers, also known as hemp hurds, are used. The bast fibers, which are tightly attached to the external cuticle and epidermis, contain the highest cellulose content, followed by hurds, consisting of 40–48% cellulose [17].

In the present study, hemp stems, acquired after seeds’ harvesting, were peeled and the collected epidermis parts together with the cuticles were previously macerated in alcohol, and then subjected to cellulose extraction by using a sustainable process, reducing to a minimum the use of strong alkali/acid solvents without compromising the final cellulose performance. The recovered hemp cellulose was esterified with citric acid, to improve its hydrophobicity, and it was embedded as reinforcement filler in a poly(lactic acid) (PLA) matrix, obtaining innovative hemp cellulose/PLA-based green biocomposite films by using a microtwin extruder coupled to a microcasting film line. Structural, thermal, and morphological characterization was performed on starting materials and the final developed biofilms.

## 2. Materials and Methods

### 2.1. Hemp Cellulose (HC) Extraction

**Figure 1:**
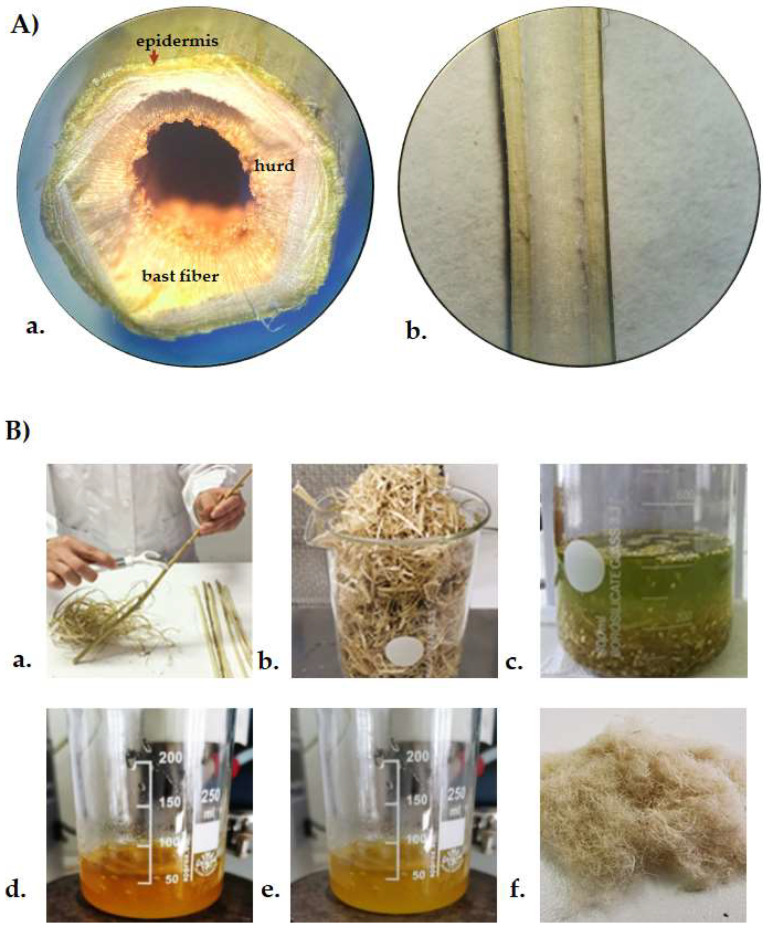
(**A**) Hemp Stem (a) transversal section; (b) longitudinal section; (**B**) Hemp cellulose extraction steps. (a) Hemp stem peeling; (b) Ultrasound-assisted maceration; (c) Alkaline treatment; (d) Acid hydrolysis; (e) Hemp cellulose recovery; (f) Representative cellulose sample.

Hemp stems (*Cannabis sativa* L. *cv*. USO31) (Figure 1A), with an average diameter of 14.6 ± 1.2 mm, were provided by Hemp Farm Lab, a local industrial hemp producer (Caserta, Italy). Stems were peeled, chopped, and sonicated in an ultrasound bath with pure EtOH at a frequency of 40 kHz (UAM, Branson Ultrasonics™ Bransonic™ M3800-E; Danbury, CT, USA) (3 cycles, 30 min each, solid:liquid ratio of 1:10 (*w*/*v*)). The obtained epidermis/cuticle material, after ultrasound-assisted alcoholic maceration, was dried at room temperature and subjected to alkaline bleaching at 50 °C for 3 h by using a 5% NaOH aqueous solution and a solid:liquid ratio of 1:10 (*w*/*v*). Acid hydrolysis was then carried out by using 10% H_2_SO_4_ (1:10 *w*/*v*) at 45 °C for 30 min, under continuous stirring. The obtained cellulose was washed until neutrality was achieved, and dried in an oven at 50 °C. The extraction yield was estimated to be 68%. The main steps followed for hemp cellulose extraction are shown in Figure 1B.

### 2.2. Hemp Cellulose Functionalization

**Figure 2:**
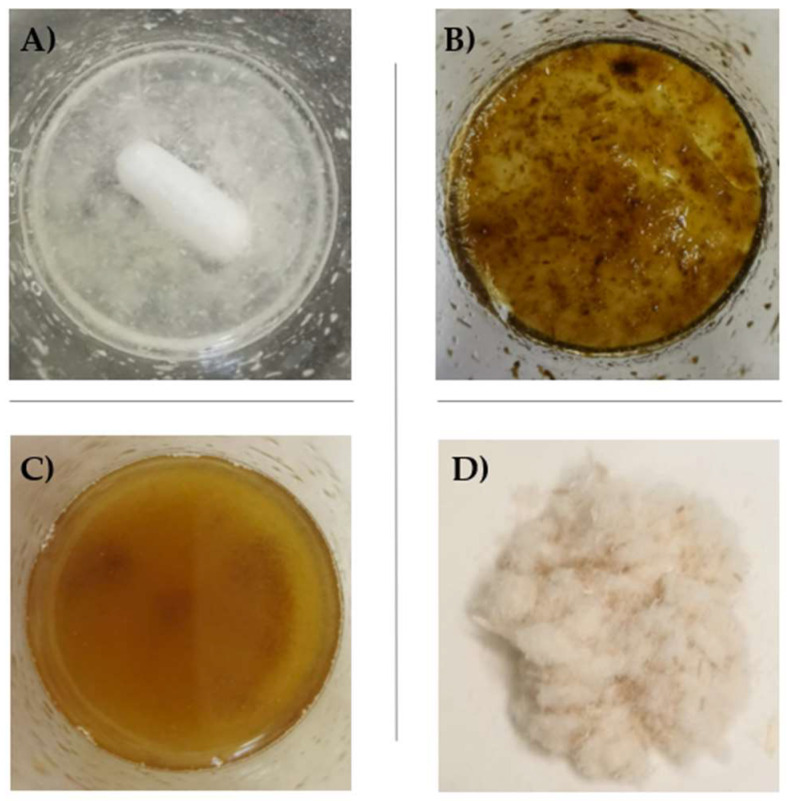
Different steps performed in HC functionalization with citric acid: (**A**) HC + water under stirring; (**B**) HC after 15 h at 130 °C; (**C**) HC after washing; (**D**) dried HC-CA obtained sample.

Hemp cellulose (HC) was esterified with citric acid (CA) at a laboratory scale, according to the protocol proposed by Cui et al. [18], with slight modifications. Briefly, 500 mg of HC were dispersed in 50 mL of distilled water under continuous stirring. Then, CA (1.5 g) was added and allowed to completely dissolve. The mixture was then kept in an oven at 130 °C for 15 h. The derivatized HC, hereinafter indicated as HC-CA, was filtered and washed with water up to pH 7 to remove the free CA excess. Finally, after sequential washing of HC-CA with methanol (50 mL) and acetone (50 mL), it was vacuum-dried (Figure 2). Structural characterization by Attenuated Total Reflection–Fourier Transform Infrared (ATR-FTIR) spectroscopy was performed to confirm structural modifications obtained after hemp cellulose citrate synthesis.

### 2.3. PLA-Based Biocomposite Films Preparation

**Figure 3:**
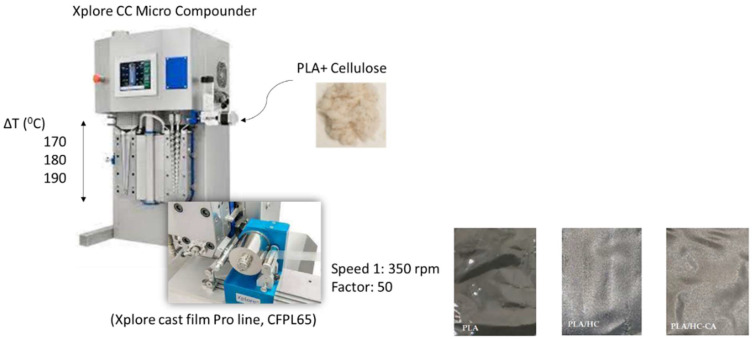
Scheme of the PLA-based biocomposite films manufacturing process performed in this work.

PLA-based biocomposite films were prepared as previously reported by Ramos et al. [19], with slight modifications. Briefly, commercial PLA pellets (PLA-4060D, 11–13%wt d-isomer, Natureworks Co., Minnetonka, MN, USA) were kept in an oven at 45 °C overnight before extrusion to prevent polymer hydrolysis. Mixing and extruding procedures were carried out (Figure 3) using a thermal profile of 170–180–190 °C at 150 rpm screw speed for 5 min by using a twin-screw microextruder (Dsm Explore 5&15 CC Micro Compounder, Heerlen, The Netherlands) combined to a cast film line (Xplore CFPL65, Heerlen, The Netherlands). HC and HC-CA samples (5 and 10%wt; Table 1) were added as organic fillers. The average thickness of films was around 23.5 μm, measured with a Digimatic Micrometer Series 293 MDC-Lite (Mitutoyo, Kyoto, Japan) at four random positions.

### 2.4. Characterization

#### 2.4.1. ATR-FTIR

ATR-FTIR spectra were recorded, in triplicate, using a Bruker Analitik IFS 66 FTIR spectrometer (Ettlingen, Germany) equipped with an ATR accessory. Films with a size of 1 × 1 mm^2^ were directly placed on the ATR crystal area. Spectra were recorded in the absorbance mode from 4000 to 500 cm^−1^, using 64 scans and 4 cm^−1^ resolution, and corrected against the background spectrum of air. Data processing was performed using OriginPro 2015 software (OriginLab Corp., Northampton, MA, USA).

#### 2.4.2. X-ray Diffraction (XRD)

XRD patterns of the developed films were recorded on a Bruker (Billerica, MA, USA) D8-Advance diffractometer equipped with a Goebel mirror for non-planar samples, a high-temperature chamber (up to 900 °C), a Kristalloflex K 760–80F X-ray generator (power 3000 W, voltage 20–60 kV and current 5–80 mA) and X-rays tube with a copper anode. Data were recorded by using Cu Kα radiation (1.5406 Å) at room temperature at scattering angles (2θ) ranging from 2.5° to 80° (step size = 0.05° min^−1^). The Segal method was used to calculate the crystallinity index of cellulose-based materials [20]. Data processing was performed using OriginPro 2015 software (OriginLab Corp., Northampton, MA, USA).

#### 2.4.3. Transmission Electron Microscopy (TEM)

The morphology of cellulose-based materials was evaluated with a JEOL JEM-1400 Plus TEM (Peabody, MA, USA) equipped with an Orius SC600 camera (Gatan, Pleasanton, CA, USA) for image acquisition at an accelerating voltage of 120 kV.

#### 2.4.4. Field Emission Scanning Electron Microscopy (FE-SEM)

The surface profiles of extracted HC and PLA-based biocomposite films were evaluated by FE-SEM (Supra 25-Zeiss, Jena, Germany) to study the homogeneity of samples as well as the influence of HC and HC-CA as organic fillers on the polymer morphology. Samples were coated with a gold layer before analysis, to increase their electrical conductivity, by using a B7341 Agar automatic sputter coater (Agar Scientific Ltd., Stansted, UK).

#### 2.4.5. Thermal Analysis

Thermogravimetric analysis (TGA) tests were performed with a TGA/SDTA 851 Mettler Toledo thermal analyzer (Mettler-Toledo S.p.A., Milan, Italy). Approximately 3 mg of samples were heated from 25 °C to 700 °C at a heating rate of 10 °C/min under a nitrogen atmosphere (flow rate 50 mL/min).

Differential scanning calorimetry (DSC) tests were carried out to determine glass transition temperature (T_g_) values in all developed films by using a TA DSC Q-2000 instrument (New Castle, DE, USA) under a nitrogen atmosphere (flow rate 50 mL/min). Samples (3 mg) were initially submitted to −90 °C in isothermal mode for 3 min. The temperature program followed consisted of a first heating from −90 to 200 °C, then cooling to −90 °C, and a further second heating to 200 °C, all these stages at 10 °C/min heating/cooling rate. Three replicates of each sample were performed.

Data processing was performed using OriginPro 2015 software (OriginLab Corp., Northampton, MA, USA).

### 2.5. Statistical Analysis

Statistical analysis of results was performed with Statgraphics Centurion XVI statistical software (Statgraphics Technologies, Inc., The Plains, VA, USA). An analysis of variance (ANOVA) was carried out. Differences between average values were assessed based on the Tukey test at a confidence level of 95% (*p* < 0.05).

## 3. Results and Discussion

### 3.1. Hemp Cellulose Extraction

Hemp stem fibers represent one of the most natural and valuable sources of cellulose, with their richness estimated to be 70–74% in the outer bast fibers [21]. Cellulose present in stem bast fibers is well embedded in a complex structure, constituted also by 15–20% hemicellulose, 3.5–5.7% lignin, 0.8% pectin, and 1.2–6.2% wax [21]. The cellulose network needs to be disrupted to improve some features, such as crystallinity and thermal stability [22]. Hemp hurds, the inner part of plant stems, have been extensively characterized as waste materials due to their cellulose content [23] for obtaining biocomposites with possible applications in green and sustainable packaging [24]. The particular stiffness and toughness properties of hemp stem fibers, which are comparable to those of flax or ramie, combined with high biodegradability and low cost, make them suitable as reinforcing materials in polymer matrix composites, e.g., instead of glass fibers [10,25]. Considering the outside protective layer, in this work, the development of a sustainable extraction process for hemp cellulose was carried out, avoiding the use of toxic solvents (e.g., toluene) and reducing NaOH and H_2_SO_4_ amounts as much as possible, without losing cellulose performance. Different chemical methods have been proposed to obtain micro- and nanocrystalline cellulose from plant matrices, involving organic solvents in the de-waxing step, followed by alkaline bleaching treatment and acid hydrolysis, to enhance the crystallinity index through the removal of amorphous regions [26]. A high sulfuric acid percentage (up to 64%) was used to obtain cellulose nanocrystals from ramie [27], as well as from other raw matrices [28,29,30,31], including hemp [32,33].

**Figure 4:**
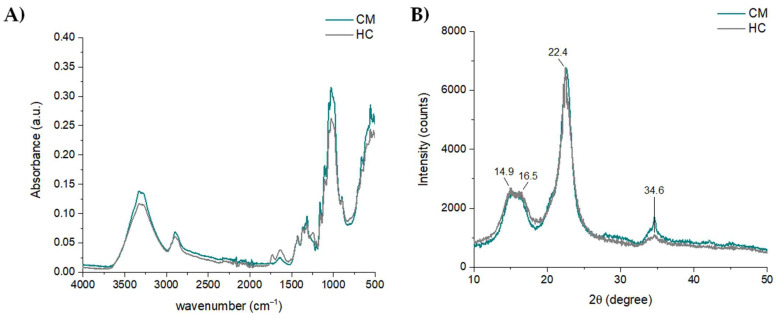
(**A**) ATR-FTIR spectra and (**B**) XDR patterns of HC sample and microcrystalline cellulose (CM) standard. Spectra were processed using OriginPro 2015 software.

In this study, hemp stems were peeled and the epidermis/cuticle obtained was sonicated with EtOH, followed by maceration with 5% NaOH, to gradually remove chlorophylls, lignin and hemicellulose, favorably recovering bioactive compounds [34]. Finally, amorphous regions were effectively reduced by using 10% H_2_SO_4_, aiming to obtain microcrystalline hemp cellulose (HC). This latter was characterized by means of infrared spectroscopy and X-ray diffraction and compared to a microcrystalline cellulose standard (CM) (Figure 4). Comparable results were obtained for HC and CM samples by using both techniques, suggesting that the isolated HC was a valuable starting material to be used for further functionalization. XRD diffractograms showed the typical diffraction planes at 2θ angles of 14.9, 16.5, 22.4, and 34.6°, in agreement with previously reported data [24,34]. The crystallinity index calculated from XRD patterns was 74%. A detailed description of ATR-FTIR data is reported in Section 3.2 and compared to functionalized cellulose.

**Figure 5:**
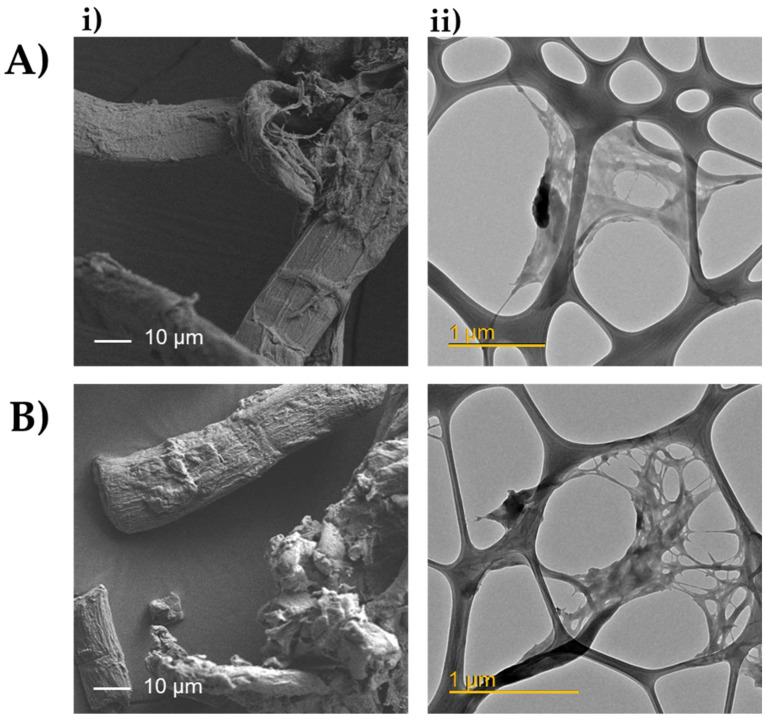
Representative FE-SEM (500× magnification) (i) and TEM (ii) images of (**A**) HC and (**B**) CM reference material.

In addition, morphological analysis performed by FE-SEM and TEM revealed a close similarity between the extracted hemp fiber cellulose (HC) and the microcrystalline cellulose standard (CM), used as reference material. In Figure 5, representative FE-SEM images at 500× magnification are reported. As it can be seen, HC appeared as isolated fibrils with a rod-like structure and an average diameter of 15 µm, suggesting that cementing wax, hemicellulose, and lignin were almost removed during the extraction procedure [35,36]. TEM analysis images revealed a large crystalline region and small quantities of amorphous regions (Figure 5). These findings are consistent with those previously explained by FTIR and XRD.

### 3.2. Chemical and Thermal Characterization of Hemp Fiber Cellulose Functionalized with Citric Acid (HC-CA)

The main disadvantage of the use of hemp cellulose as a reinforcing filler in PLA is its low compatibility, due to the poor hydrophilicity of the polymer matrix, compared to the cellulose material [37]. To overcome this drawback, the functionalization of the cellulose surface by introducing acid groups able to establish hydrogen bonds with PLA carbonyl groups can be used [38]. Different esterification agents have been proposed, such as anhydrides, although the use of carboxylic acids has been considered more environmentally friendly. In this context, citric acid has proved to be a valuable option, as it can also favor the crosslinking of cellulose chains, resulting in an enhanced polysaccharide hydrophobicity [39]. In this work, the esterification of the obtained HC was achieved by using citric acid, and the obtained HC-CA functionalized cellulose was characterized both chemically and thermally, before being added to PLA.

**Figure 6:**
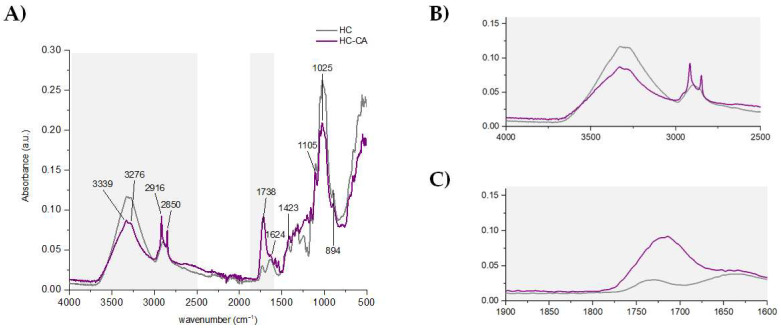
(**A**) ATR-FTIR spectra of a representative HC-CA sample; (**B**,**C**) zoomed overlapped FTIR representative spectra of HC and HC-CA samples, showing main spectral differences. Spectra were processed using OriginPro 2015 software.

ATR-FTIR analysis highlighted the main differences between native and functionalized hemp cellulose, confirming some structural modifications. In Figure 6, the FTIR spectrum of HC-CA is shown, together with two zoomed regions to better detect signals’ modifications occurring after citric acid treatment. The decrease in intensity of the broad bands appearing at 3339 and 3276 cm^−1^ was associated with a certain degree of substitution; being attributed to the stretching vibration of cellulose OH groups forming intra- and intermolecular H-bonds [40], respectively, which are more hindered as a consequence of citric acid intercalation in the saccharide chains. On the contrary, the intensity of peaks appearing at 2916 and 2850 cm^−1^, which were ascribed to C_sp3_-H stretching vibrations (both asymmetric and symmetric, respectively), increased. This behavior, together with the broad band showed in the range of 1737–1723 cm^−1^ (which was attributed to C=O stretching of both ester and citric acid free carboxylic groups) [18], confirmed the interaction hypothesis. Since samples were washed abundantly with water to remove the excess of the non-reacted acid reagent, these bands likely represent only the functionalized sample without the presence of significant interferences. The absorption bands detected in the fingerprint region at lower wavelengths (1300–800 cm^−1^) were related to the hemp cellulose matrix, as previously reported [41].

In order to evaluate the relative crystallinity index of HC-CA, the intensity ratio between the signals showed at 1423 cm^−1^ (due to the CH_2_ scissoring of crystalline cellulose) and 894 cm^−1^ (related to β-glucosidic linkage in amorphous cellulose) was calculated. It was found to be 1.2-fold higher in HC-CA than in HC [42], being very similar to that observed for CM, as previously confirmed by XRD analysis.

**Figure 7:**
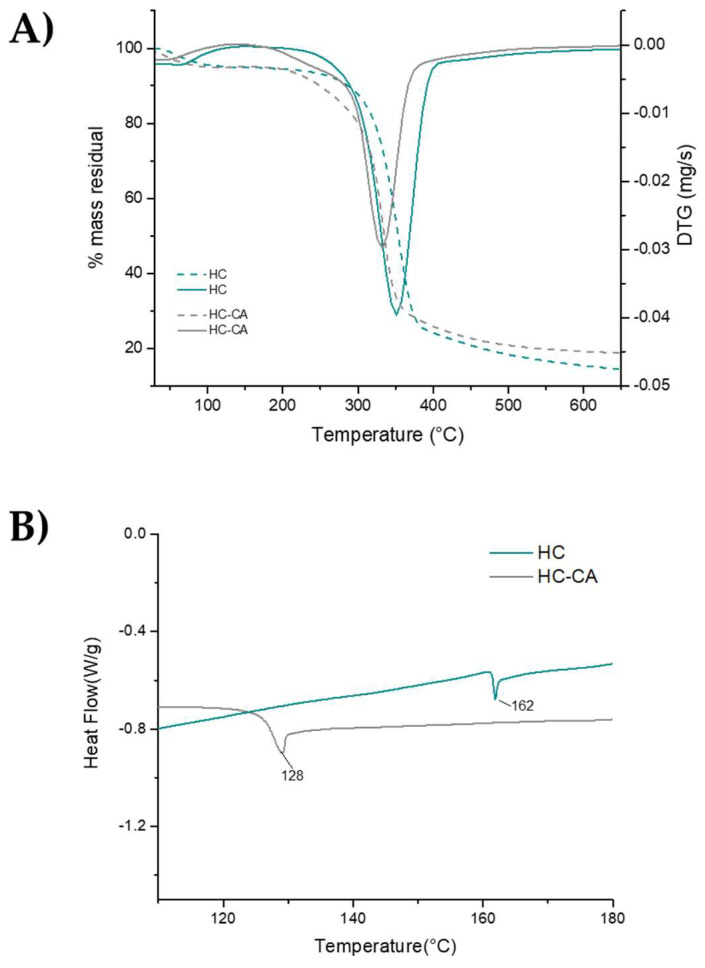
(**A**) Primary and derivative representative thermograms and (**B**) DSC curves of HC-CA and HC samples. Data were processed using OriginPro 2015 software.

The thermal behavior of the HC-CA sample before its incorporation as filler in the PLA matrix was evaluated by TGA and compared to the native HC. In Figure 7A, representative TGA/DTGA curves are shown, evidencing two weight loss steps. The first one occurred in the range of 50–90 °C (<5%) and it was associated with water evaporation. Regarding the main degradation step, significant differences (*p* < 0.05) were obtained between the analyzed samples (Table 2). The maximum degradation temperature value obtained for HC-CA was slightly lower (*p* < 0.05) compared to that of the native HC sample (Table 2), and it was associated with cellulose hydroxyl group substitution [43].

DSC curves showed a clear change in heat flow during the second heating scan, confirming the formation of ester bond linkages. Indeed, the endothermic peaks observed from 40 °C to 150 °C during the first heating (not shown) were related to water evaporation [44], whereas in Figure 7B a 34 °C lower T_g_ was found for HC-CA compared to HC, in accordance to an enhanced plasticity of the functionalized material.

### 3.3. PLA-Based Biocomposite Films: Morphological, Structural, and Thermal Characterization

HC-CA sample was embedded into a PLA amorphous resin and characterized in terms of structural, thermal, and morphological properties. For this purpose, the obtained films were compared to neat PLA and PLA/HC formulations to investigate the interactions between the polymer and the organic filler. PLA is known to exhibit several benefits, such as being compostable, cost-effective, easy to process, and non-toxic [45,46]. In addition, the Food and Drug Administration (FDA) has approved it as generally recognized as safe (GRAS) for food and pharmaceutical products. However, its low opacity, limited toughness, and thermal and mechanical features require the addition of reinforcement materials able to overcome these shortcomings [47,48].

#### 3.3.1. Morphological Analysis by FE-SEM

**Figure 8:**
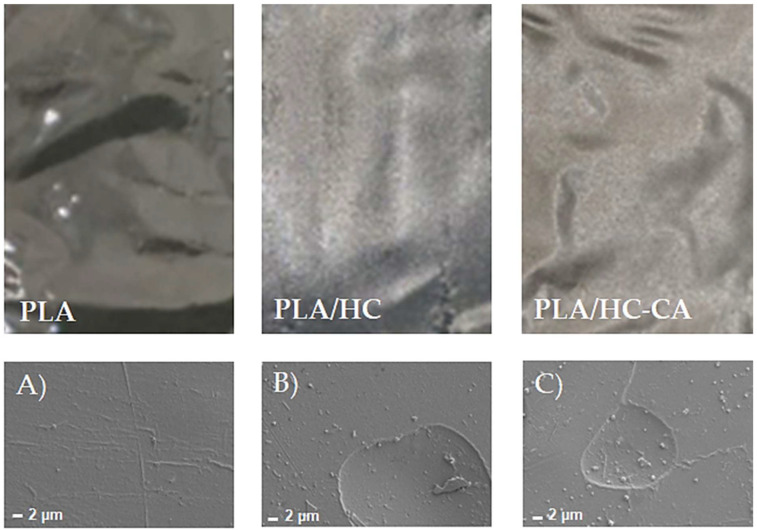
Representative pictures of PLA-based biocomposite films and their corresponding FE-SEM images (2000× magnification). (**A**) FE-SEM image of PLA film; (**B**) FE-SEM image of PLA/HC film; (**C**) FE-SEM image of PLA/HC-CA film.

Representative pictures of neat PLA and PLA-based biocomposite films as well as their FE-SEM images are shown in Figure 8. In agreement with the literature data, the neat PLA fracture surface appeared quite smooth [49], whereas PLA/HC and PLA/HC-CA samples showed small circular areas, corresponding to the organic filler dispersed in the PLA matrix. This behavior was indicative of a homogeneous composition in the biocomposite films, being hemp cellulose well integrated into the polymer matrix.

#### 3.3.2. ATR-FTIR and XRD Analyses

**Figure 9:**
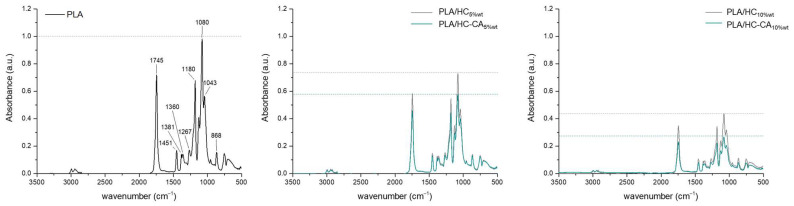
ATR-FTIR spectra of PLA and PLA-based biocomposite films. Spectra were processed using OriginPro 2015 software.

In Figure 9, ATR-FTIR spectra of PLA-based biocomposite films are shown. Overlapped spectra were observed for PLA and biocomposite films, only differing in band intensities. In particular, a decrease in the peak appearing at 1745 cm^−1^ was found, which was attributed to the symmetrical stretching of PLA C=O ester groups [47], besides other characteristic PLA bands at 1451 (ν CH_3_), 1381 (δ C-H), 1360 (δ CH_3_), 1180 and 1080 (ν C-O), and 868 (ν C-C) cm^−1^ [50]. The absence of other different bands than those corresponding to PLA suggested a physical interaction between the polymer and the organic filler [47], whereas lower intensities observed for biocomposite films could be in accordance with their chemical interaction [51]. In particular, this absorbance decrease was found to be filler amount-dependent, and within the same percentage, HC-CA was more prone than HC to interact.

**Figure 10:**
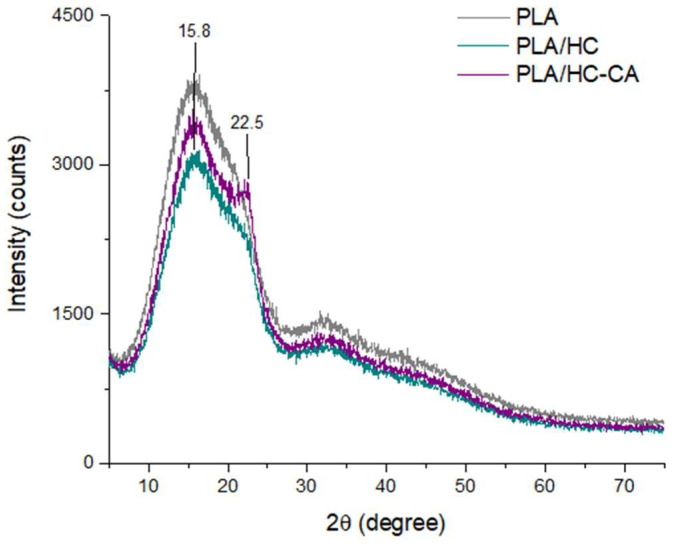
Representative XRD patterns of PLA-based biocomposite films (PLA + hemp cellulose; PLA + functionalized hemp cellulose), compared to neat PLA. Spectra were processed using OriginPro 2015 software.

A structural investigation by XRD was also performed to evaluate the effect of the organic filler in improving the crystallinity of neat amorphous PLA. Representative XRD diffractograms are depicted in Figure 10. A broad maximum peak was detected for all samples at 2θ around 16°, which was attributed to PLA according to the literature data [52]. In addition, when native hemp cellulose was embedded into PLA, diffraction peaks seemed to be masked by the polymer matrix; whereas after citric acid functionalization, a small shoulder at 22.5° was found, suggesting that the overall crystallinity of HC-CA was also reflected in the film structure after the filler incorporation [47,53].

#### 3.3.3. Thermal Analysis

**Figure 11:**
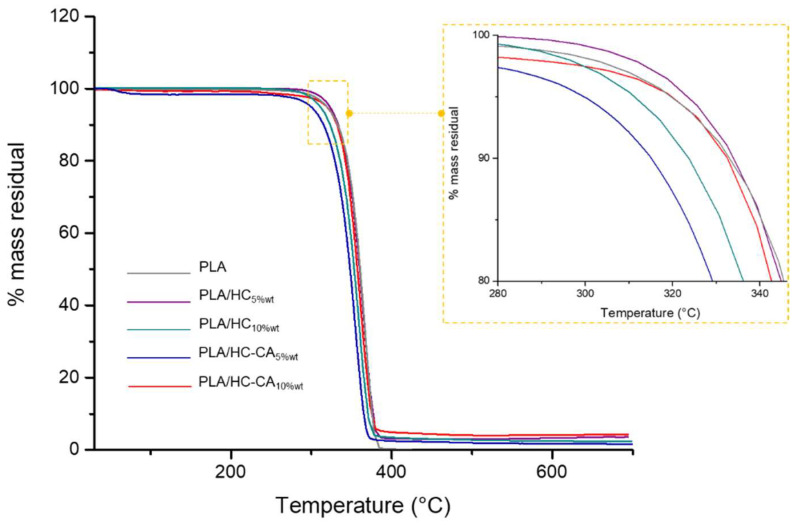
TGA representative thermograms of the studied PLA-based films. In the yellow box a zoomed region, highlighting T_ini_5__ differences, is reported. Thermograms were processed using OriginPro 2015 software.

TGA and DSC analyses were carried out on PLA biocomposite films and neat PLA, in order to evaluate potential differences in their thermal behavior due to the presence of the organic filler within the polymer matrix. In Figure 11, TGA thermograms are depicted, whereas in Table 3 the main obtained thermal parameters are shown. TGA analysis revealed a single degradation step occurring in the range of 300–400 °C for all samples. This behavior was in accordance with previous studies reporting PLA degradation peaks between 332 and 363 °C [19]. Moreover, the initial degradation temperature obtained for PLA/HC_5%wt_ was significantly (*p* < 0.05) higher compared to the other developed films, showing some increase in thermal stability caused by the addition of the cellulose filler at 5%wt content. In contrast, no statistical differences (*p* > 0.05) were observed between all formulations regarding maximum degradation temperature values, suggesting that the incorporation of HC or HC-CA fillers into PLA did not produce a significant change in its structure, under the studied conditions. These results are in agreement with DSC results, where no significant differences (*p* > 0.05) were observed between all formulations in terms of glass transition temperature (T_g_) values. In this case, neat PLA showed a T_g_ value of 57.9 ± 1.0 °C, in agreement with previously reported data [51].

## 4. Conclusions

The obtained results in this work have demonstrated that the epidermis and cuticle of hemp stems, commonly discarded in hemp supply chains and generating serious disposal concerns, represent a precious renewable source of cellulose. A greener chemical treatment, consisting of an ultrasound-assisted maceration step followed by mild bleaching/hydrolysis, was successfully applied to remove cementing wax, hemicellulose, and lignin. After functionalization with citric acid, a significant increase in crystallinity index and a decrease in T_g_ value were found compared to non-functionalized hemp cellulose. The modified cellulose was embedded into a PLA matrix by using a microtwin extruder coupled to a microcasting film line, for the development of green biocomposite films. Structural and morphological analyses suggested a physical and chemical interaction between PLA and the organic filler in films and their homogeneous composition. The thermal behavior of neat PLA was not significantly affected by the filler addition, although a slight increase in thermal stability was observed by TGA for hemp cellulose added at 5%wt. The obtained results offer new insight into the use of functionalized cellulose from hemp stem wastes for the development of innovative packaging systems, in which biomass residues will be converted into added-value products, following the so-called “bio-refinery” model. Further work will be needed to evaluate the cost efficiency and life cycle assessment of the obtained biomaterials at a larger scale for industrial applications.

## Figures and Tables

**Table 1:** Biocomposite formulations used in this work.

Acronym	Biocomposite Formulation
PLA	Neat PLA
PLA/HC_5%wt_	PLA + extracted hemp cellulose (5%wt)
PLA/HC-CA_5%wt_	PLA + hemp cellulose esterified with citric acid (5%wt)
PLA/HC_10%wt_	PLA + extracted hemp cellulose (10%wt)
PLA/HC-CA_10%wt_	PLA + hemp cellulose esterified with citric acid (10%wt)

**Table 2:** Thermal behavior of HC-CA and non-functionalized hemp cellulose (HC) samples (values are expressed as mean ± SD, *n* = 3).

Sample	T_ini_5__ (°C)	T_max_ (°C)	T_final_ (°C)	% Residual
HC	101 ± 4 ^a^	355 ± 4 ^a^	399 ± 1 ^a^	14 ± 1 ^a^
HC-CA	87 ± 3 ^b^	336 ± 4 ^b^	387 ± 1 ^b^	19 ± 1 ^b^

Different superscripts in the same column indicate significant differences between samples at a 95% confidence level.

**Table 3:** Thermal parameters obtained for HC-CA and non-functionalized hemp cellulose (HC) (values are expressed as mean ± SD, *n* = 3). Different superscripts in the same column refer to statistically different values (*p* < 0.05).

Sample	TGA			DSC
	T_ini_5__ (°C)	T_max_ (°C)	T_final_ (°C)	T_g_ (°C)
PLA	314 ± 5 ^a^	363 ± 5 ^a^	405 ± 1 ^a^	57.9 ± 1.0 ^a^
PLA/HC_5%wt_	322 ± 5 ^b^	352 ± 1 ^a^	402 ± 3 ^a^	57.4 ± 0.7 ^a^
PLA/HC-CA_5%wt_	298 ± 1 ^a^	354 ± 6 ^a^	406 ± 1 ^a^	56.5 ± 0.1 ^a^
PLA/HC_10%wt_	309 ± 1 ^a^	358 ± 1 ^a^	406 ± 1 ^a^	57.2 ± 0.2 ^a^
PLA/HC-CA_10%wt_	320 ± 1 ^a^	360 ± 1 ^a^	408 ± 1 ^a^	56.7 ± 0.8 ^a^

## Data Availability

The data are included in this manuscript.

## References

[1] Paini J., Benedetti V., Ail S.S., Castaldi M.J., Baratieri M., Patuzzi F. (2021). Valorization of Wastes from the Food Production Industry: A Review towards an Integrated Agri-Food Processing Biorefinery. Waste Biomass Valorization.

[2] Europe Moving towards a Sustainable Future. https://ec.europa.eu/info/sites/default/files/sdg_multi-stakeholder_platform_input_to_reflection_paper_sustainable_europe2030.pdf.

[3] Tsegaye B., Jaiswal S., Jaiswal A.K. (2021). Food Waste Biorefinery: Pathway towards Circular Bioeconomy. Foods.

[4] LEGGE 2 Dicembre 2016, n. 242 Disposizioni per la Promozione Della Coltivazione e Della Filiera Agroindustriale Della Canapa. (16G00258) (GU Serie Generale n.304 del 30-12-2016). https://www.gazzettaufficiale.it/eli/id/2016/12/30/16G00258/sg.

[5] Piccolella S., Crescente G., Formato M., Pacifico S. (2020). A Cup of Hemp Coffee by Moka Pot from Southern Italy: An UHPLC-HRMS Investigation. Foods.

[6] Formato M., Crescente G., Scognamiglio M., Fiorentino A., Pecoraro M.T., Piccolella S., Catauro M., Pacifico S. (2020). (−)-Cannabidiolic Acid, a Still Overlooked Bioactive Compound: An Introductory Review and Preliminary Research. Molecules.

[7] Nigro E., Crescente G., Formato M., Pecoraro M.T., Mallardo M., Piccolella S., Daniele A., Pacifico S. (2020). Hempseed Lignanamides Rich-Fraction: Chemical Investigation and Cytotoxicity towards U-87 Glioblastoma Cells. Molecules.

[8] Nigro E., Pecoraro M.T., Formato M., Piccolella S., Ragucci S., Mallardo M., Russo R., Di Maro A., Daniele A., Pacifico S. (2022). Cannabidiolic acid in Hemp Seed Oil Table Spoon and Beyond. Molecules.

[9] Salentijn E.M.J., Petit J., Trindade L.M. (2019). The Complex Interactions Between Flowering Behavior and Fiber Quality in Hemp. Front. Plant Sci..

[10] Musio S., Müssig J., Amaducci S. (2018). Optimizing Hemp Fiber Production for High Performance Composite Applications. Front. Plant Sci..

[11] Ahmed A.T.M.F., Islam M.Z., Mahmud M.S., Sarker M.E., Islam M.R. (2022). Hemp as a potential raw material toward a sustainable world: A review. Heliyon.

[12] Marrot L., Lefeuvre A., Pontoire B., Bourmaud A., Baley C. (2013). Analysis of the hemp fiber mechanical properties and their scattering (Fedora 17). Ind. Crops Prod..

[13] Dhakal H.N., Zhang Z., Faruk O., Sain M. (2015). The use of hemp fibres as reinforcements in composites. Biofiber Reinforcements in Composite Materials.

[14] Shahzad A. (2011). Hemp fiber and its composites—A review. J. Compos. Mater..

[15] Omran A.A.B., Mohammed A.A.B.A., Sapuan S.M., Ilyas R.A., Asyraf M.R.M., Rahimian Koloor S.S., Petrů M. (2021). Micro- and Nanocellulose in Polymer Composite Materials: A Review. Polymers.

[16] Stelea L., Filip I., Lisa G., Ichim M., Drobotă M., Sava C., Mureșan A. (2022). Characterisation of Hemp Fibres Reinforced Composites Using Thermoplastic Polymers as Matrices. Polymers.

[17] Stevulova N., Cigasova J., Estokova A., Terpakova E., Geffert A., Kacik F., Singovszka E., Holub M. (2014). Properties Characterization of Chemically Modified Hemp Hurds. Materials.

[18] Cui X., Honda T., Asoh T.A., Uyama H. (2020). Cellulose Modified by Citric Acid Reinforced Polypropylene Resin as Fillers. Carbohydr. Polym..

[19] Ramos M., Fortunati E., Peltzer M., Jimenez A., Kenny J.M., Garrigós M.C. (2016). Characterization and disintegrability under composting conditions of PLA-based nanocomposite films with thymol and silver nanoparticles. Polym. Degrad. Stab..

[20] Segal L., Creely J.J., Martin A.E., Conrad C.M. (1959). An Empirical Method for Estimating the Degree of Crystallinity of Native Cellulose Using the X-ray Diffractometer. Text. Res. J..

[21] Manaia J.P., Manaia A.T., Rodriges L. (2019). Industrial Hemp Fibers: An Overview. Fibers.

[22] Panaitescu D.M., Vuluga D.M., Paven H., Iorga M.D., Ghiurea M., Matasaru I., Nechita P. (2008). Properties of polymer composites with cellulose microfibrils. Mol. Cryst. Liq. Cryst..

[23] Bokhari S.M.Q., Chi K., Catchmark J.M. (2021). Structural and physico-chemical characterization of industrial hemp hurd: Impacts of chemical pretreatments and mechanical refining. Ind. Crops Prod..

[24] Momeni S., Safder M., Khondoker M.A.H., Elias A.L. (2021). Valorization of Hemp Hurds as Bio-Sourced Additives in PLA-Based Biocomposites. Polymers.

[25] Kassab Z., Abdellaoui Y., Salim M.H., Bouhfid R., Qaiss A.E.K., El Achaby M. (2020). Micro- and nano-celluloses derived from hemp stalks and their effect as polymer reinforcing materials. Carbohydr. Polym..

[26] Ouajai S., Shanks R.A. (2005). Morphology and Structure of Bioscouring Hemp Fibre. Macromol. Biosci..

[27] Kusmono, Listyanda R.F., Wildan M.W., Ilman M.N. (2020). Preparation and characterization of cellulose nanocrystal extracted from ramie fibers by sulfuric acid hydrolysis. Heliyon.

[28] Zheng D., Zhang Y., Guo Y., Yue J. (2019). Isolation and Characterization of Nanocellulose with a Novel Shape from Walnut (*Juglans Regia* L.) Shell Agricultural Waste. Polymers.

[29] Mascheroni E., Rampazzo R., Ortenzi M.A., Piva G., Bonetti S., Piergiovanni L. (2016). Comparison of cellulose nanocrystals obtained by sulfuric acid hydrolysis and ammonium persulfate, to be used as coating on flexible food-packaging materials. Cellulose.

[30] García-García D., Balart R., Lopez-Martinez J., Ek M., Moriana R. (2018). Optimizing the yield and physico-chemical properties of pine cone cellulose nanocrystals by different hydrolysis time. Cellulose.

[31] Jordan J.H., Easson M.W., Dien B., Thompson S., Condon B.D. (2019). Extraction and characterization of nanocellulose crystals from cotton gin motes and cotton gin waste. Cellulose.

[32] Luzi F., Fortunati E., Puglia D., Lavorgna M., Santulli C., Kenny J.M., Torre L. (2014). Optimized extraction of cellulose nanocrystals from pristine and carded hemp fibres. Ind. Crops Prod..

[33] Fortunati E., Armentano I., Zhou Q., Iannoni A., Saino E., Visai L., Berglund L.A., Kenny J.M. (2012). Multifunctional bionanocomposite films of poly(lactic acid), cellulose nanocrystals and silver nanoparticles. Carbohydr. Polym..

[34] Ouajai S., Shanks R.A. (2005). Composition, structure and thermal degradation of hemp cellulose after chemical treatments. Polym. Degrad. Stab..

[35] Dalle Vacche S., Karunakaran V., Patrucco A., Zoccola M., Douard L., Ronchetti S., Gallo M., Schreier A., Leterrier Y., Bras J. (2021). Valorization of Byproducts of Hemp Multipurpose Crop: Short Non-Aligned Bast Fibers as a Source of Nanocellulose. Molecules.

[36] Sair S., Oushabi A., Kammouni A., Tanane O., Abboud Y., Oudrhiri Hassani F., Laachachi A., El Bouari A. (2017). Effect of surface modification on morphological, mechanical and thermal conductivity of hemp fiber: Characterization of the interface of hemp –Polyurethane composite. Case Stud. Therm. Eng..

[37] Cui X., Ozaki A., Asoh T.A., Uyama H. (2020). Cellulose modified by citric acid reinforced Poly(lactic acid) resin as fillers. Polym. Degrad. Stab..

[38] Dhar P., Bhasney S.M., Kumar A., Katiyar V. (2016). Acid functionalized cellulose nanocrystals and its effect on mechanical, thermal, crystallization and surfaces properties of poly (lactic acid) bionanocomposites films: A comprehensive study. Polymer.

[39] Gil Giraldo G.A., Mantovan J., Marim B.M., Kishima J.O.F., Mali S. (2021). Surface Modification of Cellulose from Oat Hull with Citric Acid Using Ultrasonication and Reactive Extrusion Assisted Processes. Polysaccharides.

[40] Oh S.Y., Yoo D.I., Shin Y., Kim H.C., Kim H.Y., Chung Y.S., Park H., Youk J.H. (2005). Crystalline structure analysis of cellulose treated with sodium hydroxide and carbon dioxide by means of X-ray diffraction and FTIR spectroscopy. Carbohydr. Res..

[41] Vârban R., Crișan I., Vârban D., Ona A., Olar L., Stoie A., Ștefan R. (2021). Comparative FT-IR Prospecting for Cellulose in Stems of Some Fiber Plants: Flax, Velvet Leaf, Hemp and Jute. Appl. Sci..

[42] Troedec M.L., Sedan D., Peyratout C.S., Bonnet J.P., Smith A., Guinebretière R., Gloaguen V., Krausz P. (2008). Influence of various chemical treatments on the composition and structure of hemp fibres. Compos. Part A Appl. Sci. Manuf..

[43] Ji H., Xiang Z., Qi H., Pranovich A., Song T. (2019). Strategy towards one-step preparation of carboxylic cellulose nanocrystals and nanofibrils with high yield, carboxylation and highly stable dispersibility using innocuous citric acid. Curr. Green Chem..

[44] Hachaichi A., Kouini B., Kian L.K., Asim M., Jawaid M. (2021). Extraction and characterization of microcrystalline cellulose from date palm fibers using successive chemical treatments. J. Polym. Environ..

[45] Sangeetha V.H., Deka H., Varghese T.O., Nayak S.K. (2016). State of the art and future prospectives of poly (lactic acid) based blends and composites. Polym. Compos..

[46] Jonoobi M., Harun J., Mathew A.P., Oksman K. (2010). Mechanical properties of cellulose nanofiber (CNF) reinforced polylactic acid (PLA) prepared by twin screw extrusion. Compos. Sci. Technol..

[47] Wang Q., Ji C., Sun J., Zhu Q., Liu J. (2020). Structure and properties of Polylactic acid biocomposite films reinforced with cellulose Nanofibrils. Molecules.

[48] Aumnate C., Soatthiyanon N., Makmoon T. (2021). Polylactic acid/kenaf cellulose biocomposite filaments for melt extrusion based-3D printing. Cellulose.

[49] Yang W., Dominici F., Fortunati E., Kenny J.M., Puglia D. (2015). Melt free radical grafting of glycidyl methacrylate (GMA) onto fully biodegradable poly(lactic) acid films: Effect of cellulose nanocrystals and a masterbatch process. RSC Adv..

[50] Nim B., Sreearunothai P., Opaprakasit P., Petchsuk A. (2019). Preparation and properties of electrospun fibers of titanium dioxide-loaded polylactide/polyvinylpyrrolidone blends. Appl. Sci. Eng. Prog..

[51] Wan Ishak W.H., Rosli N.A., Ahmad I. (2020). Influence of amorphous cellulose on mechanical, thermal, and hydrolytic degradation of poly (lactic acid) biocomposites. Sci. Rep..

[52] Armentano I., Fortunati E., Burgos N., Dominici F., Luzi F., Fiori S., Jiménez A., Yoon K., Ahn J., Kang S. (2015). Processing and Characterization of Plasticized Pla/Phb Blends for Biodegradable Multiphase Systems. Express Polym. Lett..

[53] Lu F., Yu H., Yan C., Yao J. (2016). Polylactic acid nanocomposite films with spherical nanocelluloses as efficient nucleation agents: Effects on crystallization, mechanical and thermal properties. RSC Adv..

